# Heterogeneous Physical and Cognitive Health Returns to Education: Evidence from High School & Beyond

**DOI:** 10.1002/alz70860_102252

**Published:** 2025-12-23

**Authors:** Eric Grodsky, John Robert Warren, Chandra Muller

**Affiliations:** ^1^ University of Wisconsin ‐ Madison, Madison, WI, USA; ^2^ University of Minnesota, Minneapolis, MN, USA; ^3^ University of Texas at Austin, Austin, TX, USA

## Abstract

**Background:**

Are the cognitive and physical health returns to education uniform across racial and ethnic groups in the United States? Past research has been inconclusive. A few studies posit that the benefits to higher levels of education are greater for Black people than they are for White people (Barnes et al. 2011, Sherman‐Wilkins and Thierry 2019) while others point to lower returns to higher education for Black (Assari 2018a, Assari 2018b, Holmes and Zajacova 2014, Kimbro et al. 2008) and Latinx (Turra and Goldman 2007) people. Still other studies report few differences in the returns to education across racial/ethnic groups (Eng et al. 2021, Vable et al. 2018).

We evaluate the returns to education by race and ethnicity across several outcomes of relevance to ADRD. We hypothesize that variation in the health returns to education are modest and are largely accounted for by variation in the quality of the high schools’ students attend; the curriculum to which students are exposed; the standing of the colleges where they earn their degrees; the fields of their degrees; and their levels of academic achievement in high school. Past studies have mostly been unable to observe variation in either academic achievement or the quality of educational opportunities, treating all credentials as identical. If opportunities or achievements are unequally distributed across racial/ethnic groups, however, these differences may lead to the erroneous conclusion that returns to credentials are unequal.

**Method:**

We use data from the U.S. High School and Beyond (HSB) cohort study, which has followed a nationally representative sample of ∼25,500 people from high school in 1980 through midlife in 2021‐22. HSB data from the 1980s includes rich information about educational contexts, opportunities, and outcomes; early life socioeconomic and family circumstances; spatial location; and demographic group memberships. HSB data from 2021‐22 include high quality measures of a variety of cognitive and health outcomes from extensive surveys, cognitive assessments, and biomarkers. Our outcomes measures will include a global measure of cognitive functioning at age ∼60; blood pressure / hypertension; A1C levels and diabetes; history of stroke; and self‐assessed overall health.

**Result:**

None yet.

**Conclusion:**

None yet.

